# Integrated sensing and delivery of oxygen for next-generation smart wound dressings

**DOI:** 10.1038/s41378-020-0141-7

**Published:** 2020-05-18

**Authors:** Manuel Ochoa, Rahim Rahimi, Jiawei Zhou, Hongjie Jiang, Chang Keun Yoon, Dinesh Maddipatla, Binu Baby Narakathu, Vaibhav Jain, Mark Michael Oscai, Thaddeus Joseph Morken, Rebeca Hannah Oliveira, Gonzalo L. Campana, Oscar W. Cummings, Michael A. Zieger, Rajiv Sood, Massood Z. Atashbar, Babak Ziaie

**Affiliations:** 1 School of Electrical and Computer Engineering, Purdue University, West Lafayette, 47907 IN USA; 2 Birck Nanotechnololgy Center, 1205W State Street, West Lafayette, 47907 IN USA; 3 School of Material Science Engineering, Purdue University, West Lafayette, 47907 IN USA; 4 Electrical and Computer Engineering Department, Western Michigan University, 4601 Campus Drive B-236, Kalamazoo, 49008 MI USA; 5 School of Mechanical Engineering, Purdue University, West Lafayette, 47907 IN USA; 6 Indiana University School of Medicine, 980 West Walnut Street, Building R3 Room C634, Indianapolis, 46202 IN USA; 7 Department of Pathology and Laboratory Medicine, Indiana University School of Medicine, IU Health Pathology Laboratory, 350W 11th Street, Room 4054, Indianapolis, IN 46202 USA; 8 Weldon School of Biomedical Engineering, Purdue University, West Lafayette, 47907 IN USA; 9 Present Address: Shenzhen MSU-BIT University, Shenzhen, China

**Keywords:** Electrical and electronic engineering, Microfluidics, Biosensors, Electrical and electronic engineering, Microfluidics

## Abstract

Chronic wounds affect over 6.5 million Americans and are notoriously difficult to treat. Suboptimal oxygenation of the wound bed is one of the most critical and treatable wound management factors, but existing oxygenation systems do not enable concurrent measurement and delivery of oxygen in a convenient wearable platform. Thus, we developed a low-cost alternative for continuous O_2_ delivery and sensing comprising of an inexpensive, paper-based, biocompatible, flexible platform for locally generating and measuring oxygen in a wound region. The platform takes advantage of recent developments in the fabrication of flexible microsystems including the incorporation of paper as a substrate and the use of a scalable manufacturing technology, inkjet printing. Here, we demonstrate the functionality of the oxygenation patch, capable of increasing oxygen concentration in a gel substrate by 13% (5 ppm) in 1 h. The platform is able to sense oxygen in a range of 5–26 ppm. In vivo studies demonstrate the biocompatibility of the patch and its ability to double or triple the oxygen level in the wound bed to clinically relevant levels.

## Printable patches for treating tissue trauma

Paper-based sensors that measure and adjust oxygen levels within chronic wounds could provide a low-cost solution for accelerating recovery. Such persistent injuries often result from diabetes and long-term hospitalization, and treatment is largely reliant on subjective assessment by healthcare providers. Babak Ziaie of Purdue University and colleagues have developed a proof-of-concept platform for manufacturing inkjet-printed, paper-based dressings that enable close control over oxygen levels in wound tissue—an important factor in the healing process. Their design employs arrays of integrated luminescent oxygen sensors, alongside catalytic regions that can be exposed to hydrogen peroxide to rapidly generate oxygen as needed. The authors demonstrate the potential utility of this approach with both in vitro and in vivo experiments, and they envision this platform as a foundation for future multi-sensor ‘smart’ wound dressings that can be affordably mass produced.

## Introduction

Chronic non-healing wounds (e.g., diabetic foot and bed sores) impact over 6.5 million Americans per year, costs in excess of $25 billion to treat on an annual basis, and are on the rise due to increasing levels of obesity and diabetes compounded by an aging population^1,2^. Current treatments are labor intensive, expensive, and generic, relying on regular cleaning, debridement, and topical or systemic administration of antibiotics^3^. A key part of the regimen is regular replacement of wound dressings (some of which contain therapeutic/antibacterial agents)^4–8^ and the use of negative pressure wound therapy^9,10^. Often, however, the use of existing commercial dressings (e.g., alginate, hydrogels, hydro-colloids, foams, etc.) is not sufficiently effective for significantly reducing the burden of wounds.

To completely understand the efficacy and limitations of a dressing technology, it is important to be able to monitor its effects on the wound quantitatively. In order to bring wound care to the 21st century, it is important to develop more advanced dressings that can integrate sensors (pH, oxygen, and inflammatory mediators), drug/cell delivery (antibiotics, growth factors, stem cells, and oxygen), and electronic intelligence; such integration can drastically improve wound care by measuring individual responses and enabling appropriate adjustments to therapy^8,11^.

In current clinical practice, the selection of dressings and other wound management systems for specific wounds, as well as the day-to-day management of the healing progress still rely heavily on visual inspection by the healthcare practitioner^12^. Unfortunately, subjective assessments cannot always provide precise insight into the status of the wound, especially when the state of wound bed tissue (especially for larger wounds) can vary within millimeters to centimeters^13^; hence, single point measurements may not provide a sufficiently informative picture of the wound tissue biology^14,15^. To be able to provide more quantitative assessments of wound healing status (with multiple sampling points per wound), researchers have been recently investigating the use of electrochemical, chemical, and biological parameters such as pH^16,17^, oxygen^18,19^, and infection^20,21^. This approach may allow quicker and more accurate data-driven diagnoses to identify the best procedure for optimal healing. Most implementations of this approach, however, still suffer from one or more of the following critical limitations: the inability to operate in situ, lack of mechanical flexibility, use of cytotoxic materials that makes the device unusable in vivo, high cost of manufacturing, and a need for large external equipment (for stimulation or readout).

To develop a smart, multi-parameter wound healing system, it is important to address each of the many healing components, one component at a time. Among the many issues hampering chronic wound healing, suboptimal oxygenation of the wound bed is one of the most critical and treatable^19,22^. Whereas acute injuries often exhibit a sufficiently functional vascular network that provides sufficient oxygen, chronic wounds lack such organization in the vascular network and are, thus, unable to receive enough oxygen to promote healing^15,19^. Although it is known that certain levels of hypoxia may trigger vascular regeneration, the severity and depth of chronic wounds can prevent adequate regeneration, causing wound ischemia. Today, hypoxia in chronic wounds is typically addressed via hyperbaric oxygen therapy, which requires bulky equipment and often exposes large areas of the body to unnecessarily elevated oxygen concentrations that can damage healthy tissue^22–24^.

Another, more practical, approach is topical oxygen therapy (TOT) in which oxygen is provided only at the wound site (e.g., by generating it or pumping it into a dressing) at atmospheric pressure, thus reducing the risk of hyperoxia while making treatment more comfortable for patients and medical staff^25^. Various commercial systems exist (e.g., OXYGENESYS, OxyBand, EPIFLO) which provide or generate oxygen to be delivered topically; however, they suffer from one or more of the following shortcomings: high cost ($2000 per 2 weeks), inability to selectively deliver oxygen to specific wound regions (to address the hypoxic heterogeneity often found in chronic wounds), and a lack of modularity for incorporation with other wound sensing components.

As a first step toward the development of a multi-functional smart wound healing bandage, here, we show a low-cost alternative for continuous O_2_ delivery and sensing comprising of an inexpensive, paper-based, biocompatible, flexible platform for locally generating and measuring oxygen in a wound region. The platform takes advantage of recent developments in the fabrication of flexible microsystems including the incorporation of paper as a substrate^26,27^ and the use of a scalable manufacturing technology, inkjet printing (i.e., the deposition of a liquid or suspension onto a substrate via the jetting of nanoliter droplets)^28–30^. The use of paper simultaneously provides structural flexibility as well as selective filtering functionality, i.e., it allows for oxygen to pass through while preventing aqueous solutions to reach the tissue and permitting ink adhesion, unlike other hydrophobic barrier materials (e.g., Tyvek, Fig. S1).

Furthermore, using a high-resolution additive manufacturing process (e.g., inkjet printing) allows for rapid, mask-less customization of designs for accelerated dressing development as well as for mass customization^31^. Such localized delivery capability can be used to customize patches for specific individuals and different wound areas in order to optimize the use of H_2_O_2_ for regions that need oxygen the most. This integration of technologies enables the development of a low-cost wound dressing with customized, wound-specific oxygen-generating and sensing regions that allow tuning of therapy in a non-invasive manner.

## Results

The oxygenation dressing takes advantage of recent developments in the fabrication of flexible microsystems and is suitable for mass production, as it features layer-by-layer fabrication and components which can be printed via commercial inkjet/screen-printing^32^. The platform consists of a flexible microfluidic network bonded to an active parchment paper substrate. A key feature is the use of parchment paper as the primary structural/functional material. Parchment paper is a hydrophobic material by design; however, its surface energy is tunable by plasma for increased hydrophilicity and ink adhesion^33^. The natural mesh structure of paper allows the spots to be embedded with chemicals suspended in an aqueous solution.

**Fig. 1:**
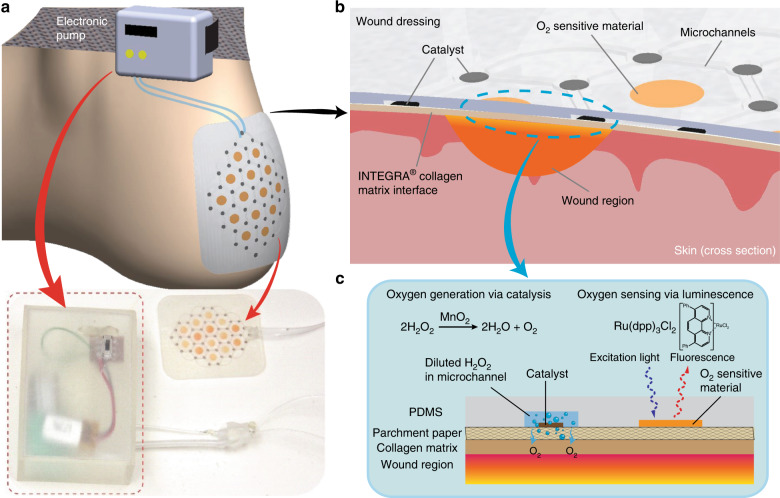
Design of the integrated oxygen sensing and delivery patch. **a** Overview illustration of the patch in use for foot ulcer applications. **b** Cross-sectional view of smart oxygen generation and sensing patch and wound area. **c** Mechanisms for generating oxygen and for sensing it for use on a flexible smart wound dressing

Figure 1 illustrates the principles of oxygen generation and sensing used in this patch. A convenient way to oxygenate wounds is to generate oxygen within the wound dressing. An economical and broadly available option is the use of hydrogen peroxide, since it is readily available in the medical environment at safe-to-use concentrations (3% v/v). A 1 mL volume of this solution contains 0.03 mL = 44 mg = 1280 µmol of H_2_O_2_, which can be catalyzed (at a stoichiometric ratio of one mole O_2_ per two moles of H_2_O_2_) to produce 640 µmol O_2_ (10 mg O_2_). At 37 °C, one mole of an ideal gas occupies about 23.4 L, thus, 640 µmol O_2_ would occupy about 15 mL. Hydrogen peroxide is a commonly used consumable due to its clean decomposition into oxygen and water by well-known materials. This feature allows it to be safely injected into aqueous systems without risk of producing unwanted contaminants. Hydrogen peroxide can be catalyzed by many transition metals and their compounds to produce oxygen via the equation below.

2H_2_O_2_ → 2H_2_O + O_2_

Of the various catalyst materials available, manganese dioxide stands out as a convenient option. Manganese dioxide micro-particles are biocompatible and catalyze hydrogen peroxide cleanly while possessing the additional advantages of being economical, simple to synthesize, and commercially available. In addition, the catalyst can be synthesized rapidly via atmospheric reduction of KMnO_4_ (accelerated by using a surfactant as part of an ink formulation) without requiring complex fabrication techniques. This process also allows straightforward deposition of the catalyst on various substrates via standard commercial material deposition inkjet printers. In this patch, oxygen delivery is achieved by flowing H_2_O_2_ into the microchannel network; when the H_2_O_2_ reaches a catalyst region, it is decomposed by the catalyst, resulting in oxygen generation (and diffusion into the wound bed) at that location^34^.

For sensing oxygen, the substrate is also patterned with a phosphorescent oxygen-sensitive ink to enable optical sensing of oxygen alongside delivery. Optical quantification techniques are ideal for sensing wound healing parameters since they allow precise measurements without unnecessarily disturbing the wound (which could hamper the healing rate). Various ruthenium- and palladium-based materials are known to exhibit oxygen-dependent phosphorescence properties^35^. Specifically, such materials can be electronically excited by illumination with light of sufficiently high energy to emit a phosphorescent response. In the presence of oxygen, the oxygen molecules can quench the material and alter the phosphorescence duration after a pulse excitation. By measuring the phosphorescence decay rate, it is possible to infer the oxygen concentration in the vicinity of the sensing material. Such techniques have been commercialized for use as optical oxygen-sensing systems. In this work, we further developed this technique by creating an oxygen-sensing ink that is based on a ruthenium compound (Ru(dpp)_3_Cl_2_). This specific compound exhibits a sufficiently strong phosphorescent response to be detected using an external optical probe. In addition, it is excitable at wavelengths in the visible range, and thus does not require high-energy light (which can affect DNA and other biological components in the wound). The ink is printable by commercial inkjet printers and ideal for use in flexible oxygen sensing applications. In particular, we have tuned its surface interaction properties with parchment paper to allow it to be printed alongside the oxygen-generation ink described above, this allowing concurrent oxygen generation and sensing in wound regions.

### Print quality

For stable drop formation and proper jetting of any ink, the *Z* number (*Z* = 1/Ohnesorge number (*Oh*))^36^, which is a dimensionless constant and a measure of density, surface tension, and viscosity should be in the range of 2–10. The *Z* number is mathematically calculated using Eq. (1).

1 $$Z = \frac{1}{{Oh}} = \frac{{Re}}{{\sqrt {We} }} = \frac{{(d\rho \gamma )^{1/2}}}{\eta }$$

where, *Re* is Reynolds number, *We* is Weber number, *d* is the nozzle diameter (21.5 µm), *ρ* is the liquid density, *γ* is the surface tension and *η* is the ink viscosity. Inks with viscosity <12 cP is desired for inkjet printing.

**Fig. 2:**
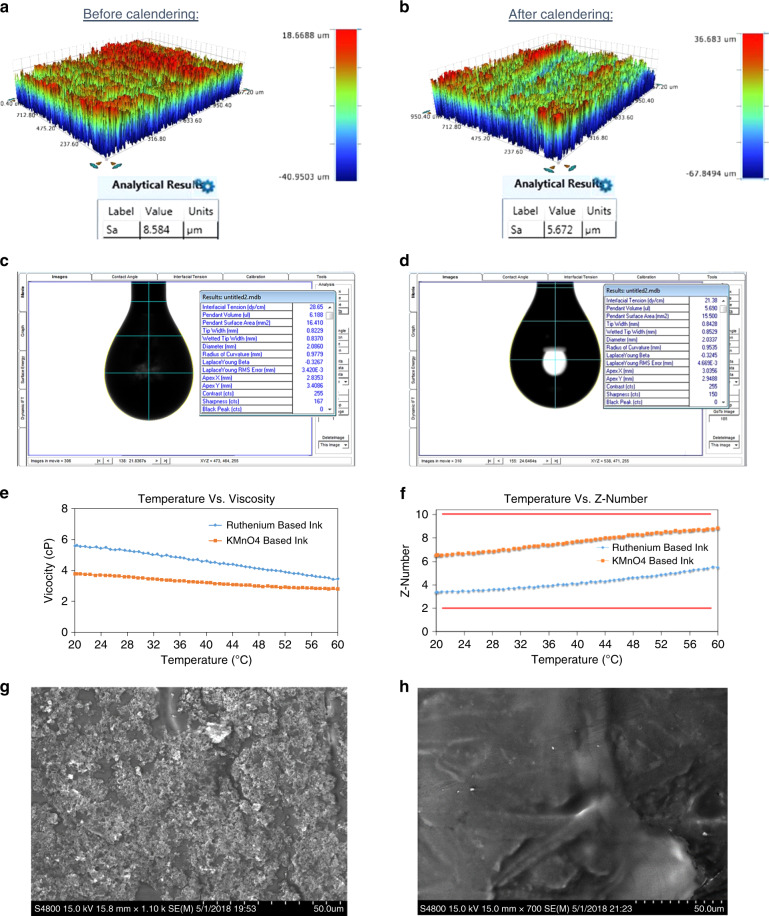
Development of functional inks. **a**, **b** Comparison of surface roughness before and after calendering of parchment paper. **c** Screen shot of FTA200 software showing the surface tension of Ru(dpp)_3_Cl_2_ based ink measured using pendant drop method. **d** Screen shot of FTA200 software showing the surface tension of KMnO_4-_based ink measured using pendant drop method. **e** Graph showing the temperature vs viscosity for Ru(dpp)_3_Cl_2_ and KMnO_4_-based inks. **f** Graph showing the temperature vs Z number for Ru(dpp)_3_Cl_2_ and KMnO_4_-based inks. **g** SEM image of the KMnO4-based ink (reduced to MnO_2_) on parchment paper. **h** SEM image of the Ru(dpp)_3_Cl_2_-based ink on parchment paper

The measured roughness of the parchment paper using Bruker Contour GT-K interferometer was 8.7 ± 1.7 µm. After calendering, the roughness of the parchment paper reduced to 5.5 ± 0.4 µm (Fig. 2a, b). This resulted in a 37% decrease in roughness, thereby resulting in a smoother substrate for printing. The measured surface tensions of the Ru(dpp)_3_Cl_2_ and KMnO_4_ ink solutions were 21.48 ± 0.12 dynes/cm and 28.28 ± 0.35 dynes/cm, respectively (Fig. 2c, d). The measured densities of Ru(dpp)_3_Cl_2_ and KMnO_4_-based ink solutions were 0.78 and 1 g/mL, respectively. The viscosity of the Ru(dpp)_3_Cl_2_ and KMnO_4_-based ink solutions decreased from 5.6 to 3.4 cP and 3.77 to 2.80 cP for the temperature range of 20–60 °C, respectively (Fig. 2e). Using Eq. (1), *Z*-numbers ranging from 3.4 to 5.5 and 6.5 to 8.8 were calculated for Ru(dpp)_3_Cl_2_ and KMnO_4_-based ink solutions, respectively, and is shown in Fig. 2f. From the results obtained, it is evident that the inks were compatible for inkjet printing at room temperature. Electron microscopy images of the two inks are shown in Fig. 2g, h; the image of the KMnO_4_ ink (Fig. 2g) demonstrates the crystalline nature of the reduced KMnO_4_, whereas the image of the Ru(dpp)_3_Cl_2_ ink (Fig. 2h) shows a smoother film coating the parchment paper fibers (compared with previously published^33^ images of parchment paper from our group). These images demonstrate the uniformity and connectedness of the ink layers printed with our inkjet printing process.

### Oxygen delivery and sensing characterization

#### Topical oxygen delivery

**Fig. 3:**
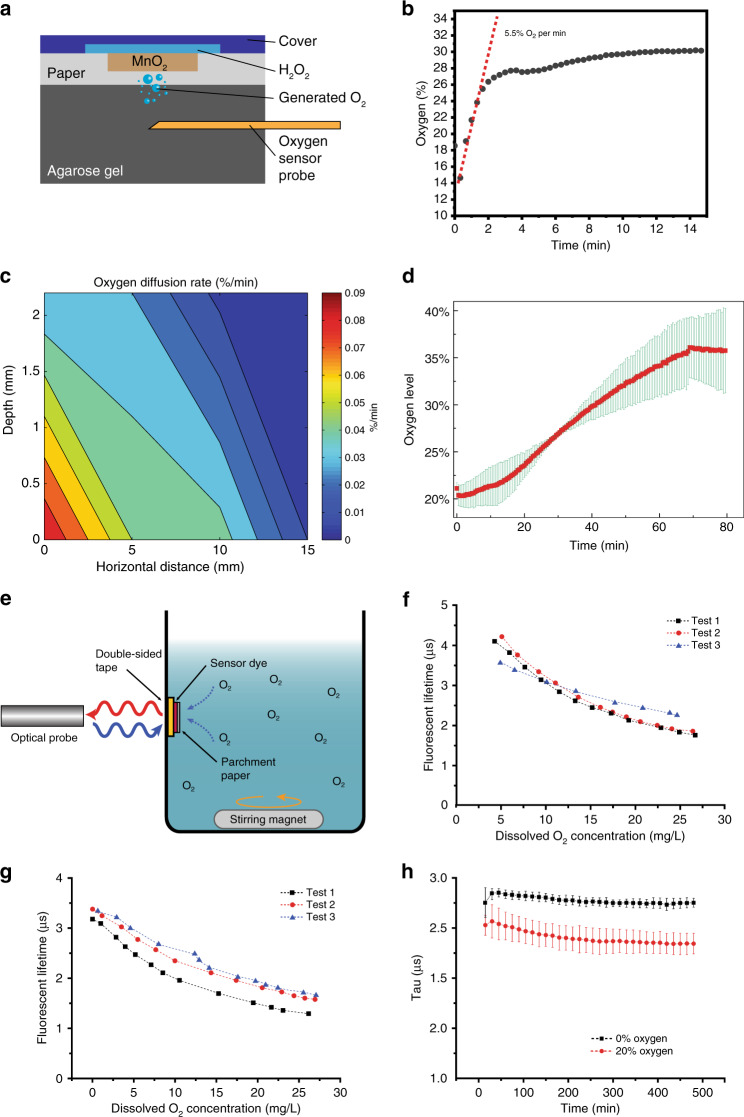
Oxygen generation and sensing characterizations. **a** Topical oxygen delivery experiment setup. **b** Oxygen delivery directly below one generation spot. **c** Gradient of oxygen diffusion from one generation spot. **d** Oxygen delivery directly below the center of one “unit cell” patch containing six generation spots. **e** Experimental setup for fluorescent lifetime measurement. **f** Fluorescence lifetime change with increased dissolved oxygen concentration from 1-layer sensing dye. **g** Fluorescence lifetime change with increased dissolved oxygen concentration from 3-layer sensing dye. **h** Fluorescence decay over time under various oxygen levels. Error bars: 1 s.d., with *n* = 3

The inkjet-printed oxygenation dye was characterized in terms of its oxygen-generation rate on agarose gel (serving as a wound phantom). The experimental techniques are detailed in the “Materials and methods” section. Briefly, open PDMS channels were closed with a substrate containing either 1 or 6 inkjet-printed spots to form a patch; the patch was placed on agarose gel, and hydrogen peroxide was pumped through the channel while the oxygen concentration in the agarose was measured using a commercial oxygen-sensing probe (Fig. 3a). When a single spot is exposed to a fixed volume of hydrogen peroxide, it can raise the oxygen concentration in agarose gel by 5.5% per minute until the peroxide is depleted (Fig. 3b).

To understand the spatial oxygen profile that can be created by a single oxygenation spot in a wound, we used the setup above (with an intermediate layer of Integra wound regeneration matrix, as would be tested in vivo), and we measured oxygen in the gel at various distances from the spot. Figure 3c shows the 3D spatial oxygen concentration by diffusing through a 0.9-mm-thick Integra into the hypoxic gel. The maximum oxygen diffusion rate is 0.09%/min (percentage per minute) at the surface of gel just below the catalyst spot (0 mm depth and 0 mm horizontal distance), while the minimum oxygen diffusion rate is 0.004%/min at the position of 2.2 mm depth and 15 mm horizontal distance inside the gel. The oxygen diffusion rate shows a normal distribution in both the depth and horizontal direction. Within the 80% area under the normal curve, the critical oxygen diffusion rate (treated as the 1/e value of the maximum) is calculated to be 0.03%/min. Therefore, the oxygen generated from a 3 × 3 mm^2^ catalyst spot can cover a range with the radius of 10 mm following the surface and the depth of 2.2 mm directly beneath it. The importance of the oxygen concentration distribution through a single oxygen-generation source is to provide an experimental baseline for designing the oxygen-generation platform with multiple sources to achieve the best efficient oxygen delivery rate for a large-scale chronic wound.

To satisfy the maximum distance requirement of 10 mm, we designed an isometric array of oxygen-generation spots to be used in oxygenation patches. The spots are separated by at most 8 mm in any direction, allowing the patch to sufficiently oxygenate the entire region it covers. To evaluate the effectiveness of using an array of spots, we tested a circular array of 6 spots (1-cm diameter “unit cell” patch) on agarose gel (with an intermediate Integra layer). The result of the oxygenation is shown in Fig. 3d; the data show a 13% increase (from 21 to 34%) over 1 h, before tapering off due to H_2_O_2_ depletion. Since the only gas generated by the patch is oxygen, the steady release rate suggests that this patch configuration can create a 100% oxygen environment (as is conventional for existing topical oxygen therapy systems) within 6 h; however, the rate can be tuned by increasing or decreasing the concentration of H_2_O_2_ used or the number of catalytic spots in the channels.

#### Topical oxygen sensing

Characterization of Tris(4,7-diphenyl-1,10-phenanthroline) ruthenium II dichloride complex (Ru(dpp)_3_Cl_2_) printed on substrate (parchment paper) was studied by measuring fluorescence lifetime under various conditions. The experiment setup is illustrated in Fig. 3e. During the experiment, oxygen gas was injected intermittently through external tubing into pre-deoxygenated water (see “Materials and methods” section for details). Oxygen concentration increase was measured using both electrochemical dissolved oxygen sensor and a Neofox® optical oxygen probe. The water was continuously stirred over time to prevent non-uniform distribution of oxygen.

As aforementioned, a method of multilayer printing was selected to increase the amount of dye being deposited. The fluorescence lifetime of printed multi-layered Ru(dpp)_3_Cl_2_ samples were firstly characterized. For this test, fluorescence lifetime (µs) was measured as a function of oxygen concentration (mg/L). Characterization for each sample was repeated three times. The result are plotted in Fig. 3f, g, for 1-layer and 3-layer printed samples, respectively. As expected, a larger fluorescence lifetime was observed in a more hypoxic condition. For 1-layer Ru(dpp)_3_Cl_2_ dye sample, fluorescence lifetime decreases from around 4 to 1.8 µs as oxygen concentration increases from 5 to 26 mg/L. And similarly, for 3-layer Ru(dpp)_3_Cl_2_ dye sample, fluorescence lifetime decreases from around 2.5 to 1.5 µs as oxygen concentration increases from 5 to 26 mg/L (5–26 ppm). Therefore, the single-layer sample showed a larger fluorescence lifetime at near zero-oxygen condition as well as a larger change of fluorescence lifetime. The data show that there is no significant difference between the sensing performance of the 1-layer and 3-layer films; thus, either can be used. However, since thinner films are preferred in wearable applications due to their superior flexibility, the 1-layer film is more appropriate for the wound-dressing application. As a result, multilayer printing of Ru(dpp)_3_Cl_2_ does not necessarily increase the performance of sensing but may degrade the uniformity and decrease flexibility.

The stability of printed Ru(dpp)_3_Cl_2_ dye was also evaluated during long-term measurements. For this measurement, the container was sealed to reduce the change of environmental oxygen outside the water. The change of the fluorescence lifetime is predominantly corresponding to the degradation of the dye. Two groups of single-layer samples were tested in both hypoxia and 20% dissolved oxygen condition. As shown in Fig. 3h, after 8 h of continuous measurements, the degradation of samples caused a variance of around 0.1 µs (~4%) in oxygen free condition and a change of 0.2 µs (~5%) in oxygenated condition. Although, due to the experiment setup, a small volume of oxygen inside the water tank may cause the reduction in fluorescence decay, the degradation of the printed sensing dye is minimal, allowing for reliable sensing over proposed operational period.

### Mechanical characterization

**Fig. 4:**
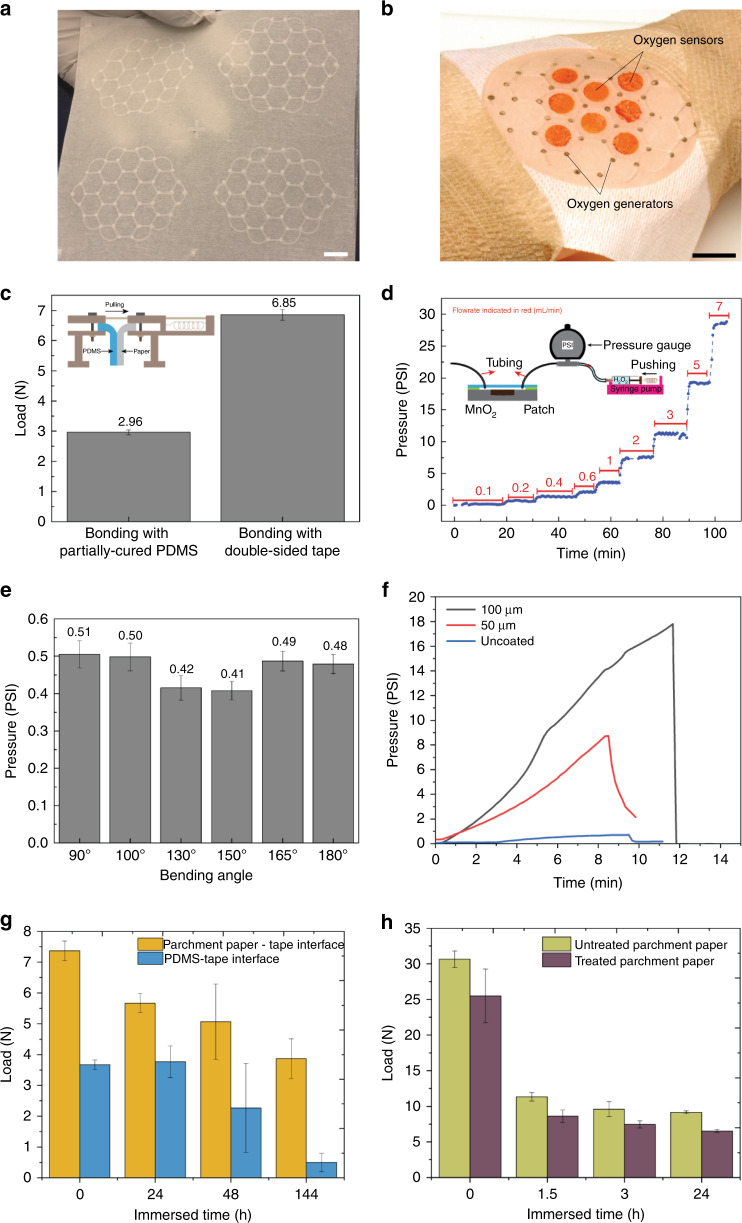
Mechanical characterization of the patch. **a** Photograph showing lamination of four patches on a single substrate. **b** Photograph showing close-up view of a single patch with oxygen generation and sensing sites. **c** Peeling strength test. **d** Bonding strength test. **e** Robustness test. **f** Increased strength upon coating the patch (on the wound side) with PDMS. **g** Effect of moisture on bond strength. **h** Effect of STERRAD treatment on material strength of wet and dry parchment paper. Scale bars: 1 cm. Error bars: 1 s.d., with *n* = 3

A key determinant of the commercial success of a novel technology is its ability to be produced in large scales. For the present wound dressing, in particular, it is important to demonstrate that the patches can be fabricated in various sizes as well as in a sheet-to-sheet fashion with multiple patches on a single sheet. The fabrication process we developed is the enabling technology for reliable scalable-volume production of the patches. Figure 4a shows these capabilities with a 2 × 2 array of 100 cm^2^ patches on a single substrate as a proof-of-concept demonstration of the scalability of the fabrication process. Figure 4b shows a close-up photograph of a patch. Visual inspection reveals uniform bonding of the layers composing the patch, and qualitative handling tests exhibited high flexibility.

To quantify the mechanical robustness of the patches, we investigated the bonding between layers and effects of flexing on the bond quality. Layer bonding was first assessed via a peel test using a universal test machine, comparing the present tape-bonding method to a previously stabilized one^37^. The patch was first cut into 2 cm wide strips and each strip was peeled several millimeters from the beginning of patch into the PDMS layer and parchment paper, each side was then fixed into the machine for the peeling test, pulling from the paper side, as illustrated in Fig. 4c; specifics of the setup are described in the “Materials and methods” section. The results show that the interface to bond PDMS and parchment paper can bear 7 N per 2 cm width, more than twice the strength of bonds using the previously established method of partially-cured PDMS as the bonding agent.

The bond strength was also tested in terms of the maximum pressure that the channels can withstand. For this test, hydrogen peroxide was pumped through a microchannel while the pressure was monitored. The results (Fig. 4d) show that the patch can withstand up to 30 PSI (207 kPa) with a flow rate of up to 7 mL/min when the outlet is left open. Pressures of up to 3 PSI (21 kPa) are possible when the flow rate is limited to 30 µL/min and the outlet is sealed (until the device fails). A typical flow rate for oxygenating a wound is on the order of 10 µL/min; therefore, the measured result approved the patch fulfills the requirement of a sustained H_2_O_2_ pumping with flow rate 10 µL/min for several hours.

The patch is designed to conform the wound, which can exhibit various degrees of curvature; therefore, the patch must reliably allow flow in the channels without leaks during pumping even when bent to fit the curvature of the wound location. To test this ability, a 100-cm^2^ patch was bent to various degrees between 90° and 180° (fully folded); hydrogen peroxide was then continuously pumped at a constant flow rate of 0.1 mL/min for 6 h while the pressure inside the microchannel network was measured and recorded. Figure 4e depicts the test setup and the results, showing a constant pressure range from 0.4 to 0.5 PSI (2.6–3.4 kPa), which indicates that the patch can sustain up to 6 h of continuous pumping even when folded completely (180°) without leakage (which would have registered as a decrease in the channel pressure). This combination of pressure and flow rate serve as a worst-case scenario demonstration of the robustness of the patch.

The dressing robustness can further be increased by applying a thin layer of an oxygen-permeable compound if needed in practice. To demonstrate the increase in robustness, wound dressings were coated with a thin layer of PDMS and tested under pressure as described above. The results (Fig. 4f) demonstrate a significant increase in the maximum pressure that the patch can withstand (up to 15 PSI) when PDMS is added. The addition of such layer would be expected to delay oxygenation, but previous studies in our group have shown that PDMS is permeable to oxygen, so the oxygen concentration on the target side of the membrane would reach the same levels after the delay^38^.

To further simulate the in vivo robustness of the wound dressing when in contact with the wound and its constituents such as serous fluid, the effect of full submergence of the wound dressing in 37 °C water on the inter- and intra-layer strength was studied as a function of time. Figure 4g demonstrates the observed inter-layer trend which shows that the PDMS-tape interface is the critical surface whose strength remains unaffected for the first 24 h, followed by successive weakening until eventual delamination on day 6. Figure 4h illustrates the impact of the sterilization process and moisture on parchment paper strength. The sterilization process leads to ~15% reduction in strength of the parchment paper and contact with water causes 60% of strength deterioration within 1 h followed by minimal changes during the subsequent 24 h.

### In vitro investigations

#### Cytotoxicity of smart dressing devices and components

The smart dressing devices and components were sterilized using a standardized low temperature hydrogen peroxide gas plasma method or STERRAD®. Although the literature suggests^39^ that electron beam or gamma radiation are effective for sterilization and tolerated by Ru-based compounds embedded in various polymers, their high cost prevented us from investigating those in these initial patches. Ethylene oxide (EO) was avoided due to environmental concerns (now supported by recent EO facility closures). STERRAD, in contrast, was conveniently available at our facilities, poses minimal concerns to personnel, and was suitable for the budget of this study.

Extracts created from STERRAD®-treated parchment paper were severely cytotoxic when compared with the extracts made from low-density polyethylene (LDPE negative control) or treatment of cells with complete growth medium for 24 h (Supplementary Fig. S2a). The cytotoxicity was reversible by washing the paper samples for 5 min in HBSS followed by 5 min in complete growth medium before extraction. Parchment paper printed with Ru(dpp)_3_Cl_2_ (1 layer) or KMnO_4_ (3 layers) was similarly non-cytotoxic when sterilized samples were washed before extraction. However, the washing steps failed to eliminate the toxicity of sterilized 3-layer (tape, PDMS, parchment paper) devices as the relative metabolic activity of cells treated with device extracts was significantly lower than that of cells treated with the extracts made from LDPE, extracts of the washed paper samples, or of cells treated with complete growth medium.

The morphological findings (Fig. S2b, S2c) were in agreement with the calculations of relative metabolic activity. The maximum number of attached cells following treatment with complete growth medium or LDPE extract was about 85–86% and indicative of slight to no reactivity. This is likely an underestimation of cell viability as some of the cells that were counted as “round” appeared as doublets, which are indicative of recent cytokinesis prior to reattachment and spreading onto the surface of the culture plate.

Extracts of unwashed paper were moderately reactive. Extracts of washed paper, including samples containing printed Ru(dpp)_3_Cl_2_ or KMnO_4_, showed only mild reactivity. Device extracts, however, caused severe reactivity despite the washing steps. Both the tape and PDMS layers used in the devices were previously shown to be non-cytotoxic.

The efficacy of passive benchtop aeration to reduce cytotoxicity was examined as an alternative to the washing protocol. Devices treated with STERRAD® followed by 7 or 14 days of passive aeration eliminated the cytotoxicity as determined by WST-1 assay and morphology (Fig. S2d). Residual cytotoxicity remained after 1 day of passive aeration and was reversible by additional washing steps. The complete reversal of the cytotoxicity using 1–2-week aeration implicates the STERRAD® process as the main source of the cytotoxicity rather than the materials used in the device.

Some sterilized, aerated devices were perfused with H_2_O_2_ for 60 min before extraction to determine if there was any added cytotoxicity associated with H_2_O_2_ perfusion and oxygen generation of functioning devices (Fig. S2e). Measurements of relative metabolic activity and morphology showed that there was significant cytotoxicity associated with 2 out of 4 perfused devices. Devices that were not perfused or had the H_2_O_2_ flushed out with 200 µL of culture medium were non-cytotoxic. This suggests that residual H_2_O_2_ associated with perfusion of the device and not the device itself is a potential source of cytotoxicity.

### In vivo investigations

**Fig. 5:**
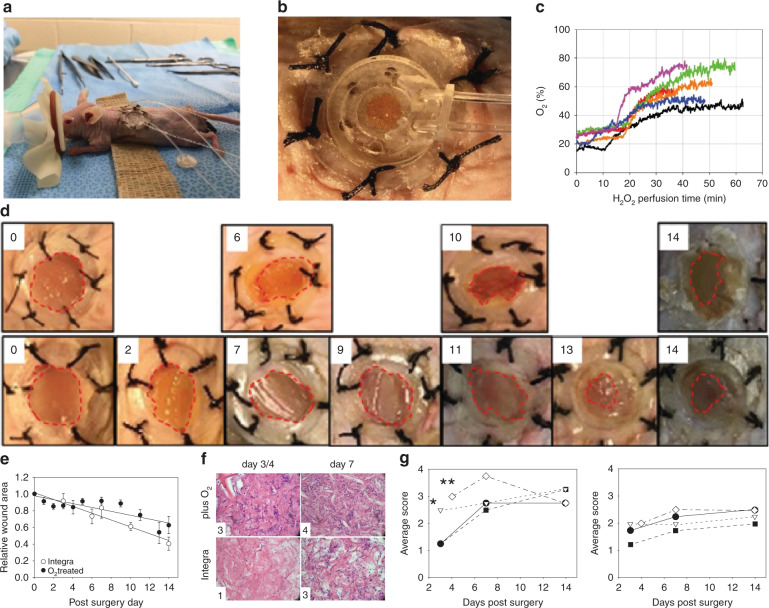
Smart dressing device testing in vivo. **a** Surgical setup. **b** Close-up of device during H_2_O_2_ perfusion showing the generation of oxygen bubbles. **c** Wound oxygen readings made during H_2_O_2_ perfusion of devices in vivo. **d** Progression of wound healing in SKH1 mouse; days 0, 2, 7, 9, 11, 13, and 14 in the oxygenated wounds and days 0, 6, 10, and 14 in the Integra control. **e** Measurements of average wound areas over time; equations are area = 0.980 ± 0.024 – (0.023 ± 0.004) × days for O_2_-treated mice (*n* = 12) and area = 1.011 ± 0.038 – (0.041 ± 0.005) × days for Integra controls (*n* = 10). **f** Hemotoxylin and eosin staining of biopsies of wounds treated with or without daily O_2_ using the device. Images show infiltration of the wound matrix by inflammatory cells. Numbers in lower left corner are inflammation scores; bar = 100 µm. **g** Assessment of Acute Inflammation (left) and Chronic Inflammation (right) of wounds following treatment with Integra alone (closed circles) or Integra plus devices containing Ru(dpp)_3_Cl_2_ ink (inverted triangle), potassium permanganate ink (closed squares) or both (open diamonds). Only the latter were perfused for 30 min with H_2_O_2_ to generate O_2_. Possible scores are 0 = none, 1 = low cell numbers, 2 = some cells, 3 = cells all over, and 4 = high numbers of cells; *p < 0.05, **p < 0.01 vs. Integra alone

The function, biocompatibility and wound healing efficacy of the devices was tested in mice. Bilateral full-thickness cutaneous wounds were created on the back of the mice and wound splints were used to prevent wound contraction, which is the normal mechanism of wound healing in mice. A collagen wound matrix was used to fill the wound defect and provide a substrate for the ingrowth of cells. The devices were calibrated for O_2_ measurements and connected to a syringe pump containing 3% H_2_O_2_ immediately before placement on the wounds (Fig. 5a). H_2_O_2_ was perfused through the devices at 200 μl/h and flow over the KMnO_4_ spots produced a vigorous generation of O_2_ as seen by the formation of gas bubbles within the device channel (Fig. 5b). Wound measurements showed that there was an initial equilibration period in which wound oxygen increased 0–5% above ambient in the first 10–20 min (Fig. 5c). In the subsequent 40–50 min, oxygen concentrations increased more sharply an additional 25–45%. Oxygen treatments were repeated daily or every other day for up to 60 min using new devices, and digital photos were taken of the wounds for wound area calculations. Figure 5d, e shows that the wound healing rate of oxygenated wounds was somewhat slower than the wounds that were not oxygenated, likely due to penetration of the paper barrier and reaction with the wound bed by H_2_O_2_ during perfusion.

Acute and chronic inflammation was assessed in the oxygenated wounds of mice up to 14 days following surgery and compared with wounds treated with wound matrix alone or wound matrix in contact with devices containing Ru(dpp)_3_Cl_2_ or KMnO_4_ ink that were not perfused. Wounds that were oxygenated using the device showed neutrophils throughout by day 4 post-surgery (Fig. 5f, g left). This response was significantly greater than the response seen in wounds containing Integra alone. The acute inflammation in treated wounds was greater after 7 days, with high numbers of neutrophils, but the response declined thereafter. Wounds covered with Integra + Ru(dpp)_3_Cl_2_ ink-containing devices also showed a stronger acute inflammatory response 3 days after surgery than wounds treated with Integra alone. The differences between all treatment groups, including Integra alone, were no longer significant by day 7 or 14 as all wounds contained neutrophils throughout. The inflammatory response in rodents to Integra typically decreases around 2 weeks after implantation^40,41^. All treatment groups showed some macrophages throughout the wound bed but there were no significant differences in the numbers between device or H_2_O_2_ perfusion groups and Integra alone (Fig. 5g, right). Similarly, the number of giant cells and granulomas were negligible in all samples.

## Discussion

The work presented in this manuscript serves as a proof-of-concept demonstration of an integrated low-cost, mass-producible wound dressing for simultaneous generation, delivery, and sensing of oxygen in the wound bed. The materials and fabrication process have been carefully developed to allow the creation of a biocompatible wound dressing with multiple functions in a commercially-scalable manner. Specifically, the functional inks developed for sensing and generating oxygen allow single-step patterning of each material without the need for masks, as is common in traditional microdevice fabrication processes. The inks have been engineered to be printable by an inkjet process such that scale-up production can leverage on established commercial inkjet manufacturing.

The characterizations of the oxygen-sensing subsystem show a reliable monitoring of oxygen via a non-invasive manner, and oxygen-generation rates are tunable and comparable to levels which affect wound healing. A similar level of oxygenation (0.3 µL O_2_/min/mm^2^) has been previously shown to effectively promote epithelial healing in a rabbit ear wound model^42^; furthermore, existing wound oxygenation systems (e.g., EPIFLO) provide oxygen flow rate in this range. Thus, this platform can generate oxygen at a sufficiently high rate to alter the oxygen level in the microenvironment of a wound, which may improve wound healing. Although the platform may require regular replacement (with an optionally updated catalyst pattern) throughout the duration of therapy, its replacement schedule (no more than once per day) is no more burdensome than common wound dressings, while serving as a platform of sensors (in this case only oxygen, but adaptable to other chemical sensors fabricated with the same manufacturing technologies). The rate of oxygenation can be further controlled by varying the amount of catalyst deposited on the spots and/or the flow rate and concentration of H_2_O_2_. Future development will focus on practical packaging measures necessary for clinical use. These include its incorporation into a commercial wound dressing as well as the implementation of an on-board hydrogen peroxide source. The microfluidic structure provides a convenient location for encapsulating H_2_O_2_ in a small (1–10 mL) pre-pressurized chamber that delivers a continuous flow through the microchannels. For example, a reservoir of dimensions 100 mm × 100 mm × 1 mm would contain enough 3% H_2_O_2_ solution to generate about 100 mL O_2_, hence enabling a production rate of 100 mL O_2_/h for up to 33 h. The peroxide concentration and/or reservoir dimensions can be adjusted to optimize for platform size or oxygenation capacity. Similar microfluidic structures (with applications as insoles) have also been previously shown to withstand the weight of a person standing or walking on them^43^, similar to the conditions that a wound dressing on the sole of a foot would experience.

In vitro and in vivo cytotoxicity studies have revealed that the materials used for fabricating the patch result in a biocompatible platform (any incompatibilities were due to hydrogen peroxide leakages, which can be readily addressed in subsequent optimization for manufacturing). In particular, measurements of relative metabolic activity and morphology showed that there was significant cytotoxicity associated with 2 out of 4 perfused devices. Devices that were not perfused or had the H_2_O_2_ flushed out with 200 µL of culture medium were non-cytotoxic. These results suggest that H_2_O_2_ is cytotoxic to cells in vitro; however, additional studies (of the patch and of H_2_O_2_ itself) are needed to conclusively determine the effects of residual H_2_O_2_ on wounds.

Similarly, in vivo results show that the patches exhibit similar inflammatory response to using an established wound regeneration matrix (Integra). Thus, for the short time period of these experiments, the patches did not increase the wound closure rate compared with Integra patches. The inflammatory response, although initially significantly greater than that in response to Integra, is not significantly different from it after 14 days. For wound healing, an inflammatory response (as well as a controlled level of ROS) may not be detrimental to healing, and it may, in fact, be beneficial^44^. More extensive studies would be needed for conclusive in vivo results, especially regarding efficacy.

Although the scope of this work is primarily the engineering of a manufacturing process for enabling the fabrication of advanced wound dressings using scalable manufacturing techniques, our budget permitted initial in vitro and in vivo biocompatibility studies. The in vivo tests conducted here are a first step to verify biocompatibility, and they are not meant to evaluate efficacy; investigations of efficacy will require additional, and more extensive in vivo tests with wound models that more adequately represent the specific application (e.g., using human fibroblasts instead of murine cells). Future studies should also investigate the impact of the dressing on the bacterial population within a wound. Since dressings often provide a warm, moist, and undisturbed environment that allows bacteria to thrive and form biofilms, the same may be true of this one; however, the release of oxygen and elevated oxygen concentrations in the wound bed may help reduce the proliferation of certain bacteria, including *Staphylococcus aureus*, as recent studies suggest^45,46^.

To ensure low cost, the design of patches like those presented here should include considerations about the fabrication process to be used (i.e., design for manufacturing). A key aspect of the design is to maintain a flat fabrication process that enables sheet-to-sheet manufacturing. For flexible hybrid electronic systems and patches like the one presented here, designing for large volume manufacturing (i.e., millions of patches per year) using roll-to-roll or sheet-to-sheet manufacturing equipment can minimize fabrication costs (due to their being continuous processes as well as additive for low material waste)^47^. Once optimized, fabrication of such systems may be marginally more complex than other topical delivery systems, but the system would provide additional value (i.e., with sensing features, especially at multiple points per wound) which other systems do not offer.

The wound dressings developed in this work present a novel integrated system for sensing and delivery of oxygen in a form factor that is amenable to commercialization. The process consists of laser-etching channels and microfluidic ports onto a PDMS-adhesive tape bilayer and subsequently laminating this onto a substrate containing inkjet-printed active inks. Connections to tubing for use with pumps and other microfluidics can be achieved by manually inserting tubing into the ports (sufficiently snug to prevent leakages). The process is currently limited to letter-size paper sheets due to the printing area of the benchtop printer used; however, the process is sufficiently robust that it can be scaled up for larger-volume production. Thus, the wound dressings present not only an advancement in scientific capabilities, but also one in manufacturing to pave the road for their commercial success.

## Materials and methods

### Print quality

#### Materials

Tris(4,7-diphenyl-1,10-phenanthroline) ruthenium(II) dichloride from Alfa Aesar® was used as the oxygen sensing dye. Potassium permanganate (KMnO_4_) from Sigma Aldrich® Chemical Company was used as the oxygen-generation catalyst. ETHOCEL™ (ethylcellulose polymer) and ECOSURF™ from Dow® Chemical Company was used as the binder and nonionic surfactant, respectively. Three percent hydrogen peroxide (H_2_O_2_) was used for oxygen generation.

#### Calendering of parchment paper

A calendering process, which uses steel rollers to compress a substrate under controlled pressures (0–40 PSI), was employed to obtain a consistent and flat paper surface. A custom made calendering machine (The Wheeler Roll Co.) was used to calender both sides of the paper substrate, with an applied pressure of 35 PSI. The roughness of the parchment paper was measured for three different samples using a Bruker Contour GT-K interferometer.

#### Ink preparation and characterization

Ruthenium-based ink was prepared by mixing Ru(dpp)_3_Cl_2_ dye and ETHOCEL™ in ethanol (solvent) in a 1:1:100 (w/w/w) ratio. The mixing was performed on a hotplate with magnetic stirrer and stirred at 700 rpm for 20 h, at room temperature. It is known that when KMnO_4_ reduces to manganese dioxide (MnO_2_) and is treated with H_2_O_2_, oxygen is generated^34,48,49^. KMnO_4_ ink solution is prepared by mixing KMnO_4_ with deionized (DI) water (15.8 g KMnO_4_/1 L H_2_O) on a hotplate with magnetic stirring at 100 rpm for 3 min, at room temperature. To make the KMnO_4_ ink solution inkjet printable, its surface tension should be decreased (<33 dynes/cm) with the help of nonionic surfactants. ECOSURF^™^ was chosen as a suitable surfactant because of its low viscosity (30 cP at 40 °C), low surface tension (29 dynes/cm at 25 °C), non-toxicity, bio-degradability, and solubility in aqueous ink systems. ECOSURF^™^ surfactant was added to the KMnO_4_ ink solution in a 1:100 (w/w) ratio for formulating the ink.

#### Ink printability characterization

The surface tension of the ink was measured by pendant drop method using a FTA200 surface tension/contact angle analyzer. A 10 mL syringe, with 1 mL of ink, was loaded into the FTA200 and the surface tension measurements were taken for three samples. An AR 2000 rheometer from TA® Instruments was used to determine the viscous behavior of both the ink solutions. Seventeen milliliters of the ink was loaded into the rheometer and the viscous behavior of the ink was obtained for 1 sample for temperatures ranging from 20 to 60 °C while the shear rate was maintained at 1000 (1/s). These values were used to compute the Z number for determining printability.

#### Inkjet printing

Multi-layered samples (1–3 layers) of the KMnO_4_ as well as Ru(dpp)_3_Cl_2_ inks were inkjet printed onto the parchment paper in an array of circular spots with a diameter of 1 and 7.5 mm, with 10 µm drop spacing and resolution of 2540 dpi, using a FUJIFILM Dimatix^™^ material deposition inkjet printer (DMP 2831). In order to achieve smooth printing, any large particles that may have agglomerated in the ink solutions were filtered using a 25 mm disposable Whatman® syringe filter, with a poly vinylidene difluoride filter (PVDF) filter membrane of 0.45 and 0.2 µm pore sizes for the Ru(dpp)_3_Cl_2_ and KMnO_4_ inks, respectively. The inks were loaded separately into Dimatix^™^ DMC-11610 cartridges (10 pl). Each layer of the Ru(dpp)_3_Cl_2_ and KMnO_4_ printed inks were cured on the platen of the inkjet printer at 55 and 60 °C, respectively.

Custom waveform patterns, which send electronic pulses to the piezo jetting device of the inkjet print-head, were developed for the Ru(dpp)_3_Cl_2_ and KMnO_4_ inks. These pulse signals control the formation, shape and ejection of the ink drops at the nozzles. Firing voltages of 40 and 30 V were applied at 4 and 5 kHz jetting frequencies for inkjet printing the Ru(dpp)_3_Cl_2_ and KMnO_4_ inks, respectively. The settings for different parameters used for the custom waveforms are shown in Table S1. The jetting waveform, with four segments, provides pulse signals to firing nozzles in order to form and eject the fluid drops (segment 1 draws ink fluid into the pumping chamber, segment 2 (firing segment) provides energy to fire the drop, segment 3 (dampening/recovery segment) and segment 4 (stand by) are designed to prevent the suction of air back into the chamber and prepare the chamber for loading the next fluid drop). The non-jetting waveform provides pulse signals to the print-head during idle/non-printing times to maintain a fluidic motion in the nozzle and prevent the drying of the ink. Each segment associated with the jetting and non-jetting waveforms are defined and controlled by various parameters such as level (%), slew rate, duration, and jetting frequency which correspond to the percent of voltage amplitude, the slope of voltage ramp which controls how fast the piezo-electric membrane is bent and the duration for which the membrane stays in the bent position, respectively.

#### Print quality characterization

Print quality was assessed by inspecting the printed samples with optical and scanning electron microscopy.

### Oxygen delivery and sensing characterization

#### Oxygen delivery characterization

Topical oxygen generation and delivery was first evaluated using the new patch and catalyst. The experiment setup is shown in Fig. 3a where a single cell (10 × 10 mm^2^) is created with one catalyst spot (1-mm diameter) and put on top of a 2 mL agarose gel within acrylic chamber (10 × 10 × 20 mm^3^), then 3% H_2_O_2_ is pumped continuously through the microchannel with the flow rate of 5 µl/min. The oxygen concentration was monitored at 0-mm depth below the catalyst spot.

To evaluate the ability to increase the oxygen concentration in the wound bed, oxygen diffusion was investigated on a surrogate wound bed, a sample of 0.3% agarose gel. An acrylic chamber with open top was assembled to hold the agarose gel sample (Fig. 3a). The chamber includes an array of 2 mm holes on one side wall to allow insertion of an oxygen probe. Prior to testing, 0.3% agarose gel was prepared and stored in a hypoxic environment until ready for use. During testing, the agarose gel was placed in the chamber. An oxygenation platform was constructed by bonding parchment paper to PDMS patterned with a chamber (3 × 3 × 2 mm^3^) and a channel (18 × 12 mm^3^). The region within the chamber was a 3 × 3 mm^2^ catalyst spot (deposited as described above). The chamber was filled with 30% H_2_O_2_ through the guide channel using a syringe pump to begin oxygen generation.

The platform was placed on top (in contact with) of the gel. The test chamber was then sealed with Parafilm barrier to prevent significant oxygenation form the atmosphere. The same oxygen probe described above was then inserted into a hole of the test chamber, penetrating the gel until the tip was positioned 3 mm directly below the catalyst spot of the paper (Fig. 3a). For this test, however, the probe was covered with a protector needle to prevent mechanical damages to the probe during insertion. The remaining holes in the chamber were sealed with adhesive tape to prevent oxygen diffusion from the atmosphere. The oxygen concentration in the gel was monitored over time.

In clinical applications, the oxygenation platform is expected to have an interfacial material between the parchment paper and the wound to create intimate contact with the wound bed. To simulate this, we repeated the above experiment with a commercial dermal regeneration matrix (Integra, Integra Life Sciences Corp.) as the interface. Integra wound matrix is 900-µm thick and is composed of cross-linked bovine tendon collagen and glycosaminoglycan that is indicated for the treatment of acute and chronic wounds, including diabetic skin ulcers. A 1 cm × 1 cm sample of Integra was cut with a razor blade and sandwiched between the oxygenation platform and the agarose gel. The rest of the experiment proceeded as above. As a control experiment, this test was repeated with empty microfluidics (i.e., no H_2_O_2_).

To investigate the range spatial effect of an oxygenation spot on a gel substrate, the oxygenation experiments were repeated for multiple locations, and the rate of oxygenation was plotted as a function of both vertical and horizontal distance from the generation spot.

#### Oxygen delivery and sensing characterization setup

In vitro validation of printed sensing material was conducted in a water container. On the side wall of a water container, inkjet-printed Ru(dpp)_3_Cl_2_ dye on parchment paper (diameter = 7.5 mm) was bonded using double-sided tape. The container and adhesive were both selected to be transparent for external fluorescence detection. An optical oxygen-sensing probe was placed pointing to the sensor at 2 mm away from the container while an electrochemical oxygen-sensing probe was submerged inside the water.

### In vitro experiments for cytotoxicity

Smart dressing devices and the parchment paper component were evaluated for cytotoxicity using the International Organization of Standardization standards 10993-5 and10993-12. The samples were placed into plastic culture dishes and sealed in Tyvek pouches for sterilization by the STERRAD® process (low temperature hydrogen peroxide gas plasma). Parchment paper, which was <0.5-mm thick, was extracted for 24 h/37 °C in complete growth medium (Eagle’s minimum Essential medium + 10% horse serum + 100 IU/mL penicillin + 100 µg/mL streptomycin) using an extraction ratio of 6 cm^2^/mL. An extraction ratio of 1.25 cm^2^/mL was used for the devices, which were more than 1.0-mm thick. In some experiments, the sterilized samples were washed in Hank’s balanced salt solution (HBSS) and EMEM or were aerated on the benchtop for up to 2 weeks before extraction. In addition, some devices were perfused with 3% H_2_O_2_ for 60 min before extraction to determine if cytotoxicity increases in functioning devices. At the time of the extraction, L-929 mouse fibroblast cells (NCTC clone 929: CCL 1, American Type Culture Collection, Manassas, VA, USA) in passage 3–10 were lifted from a culture flask using trypsin/EDTA. An aliquot was counted using trypan blue and cells were resuspended in complete growth medium at a density of 1 × 10^5^ cells/mL. Cells were dispensed into wells of 96-well culture plates (1 × 10^4^ cells/well) and cultured at 37 °C in a humidified atmosphere of 5% CO_2_/95% air. After 24 h, the culture medium was removed and replaced with 100 µL of extractant. Some wells received sodium dodecyl sulfate (0–400 µM in EMEM; positive controls), low-density polyethylene extract (1.25 cm^2^ LDPE/mL EMEM; negative control) or complete growth medium alone. Cells were then cultured for an additional 24 h. Images (mag. of 100 and 200×) of cell cultures were recorded by photo microscopy before and after treatment using an Olympus CK40 inverted microscope and Insight2 SPOT camera (Diagnostic Imaging) and the numbers of attached (live) and round or loosely attached dead cells were manually counted at a later time. Subsequently, cells in culture plates were washed once with HBSS and metabolic activity was measured by incubating cells with 100 µL of WST-1 cell proliferation reagent (Roche Diagnostics) for up to 4 h at 37 °C. To determine cytotoxicity, the absorbance of the medium in wells was measured at 450 nm after 2 and/or 4 h using a microplate reader (PHERAstar) and was corrected using absorbance measurements at 630 nm and using blanks. Values were then plotted relative to the values obtained using cells cultured in adjacent wells with complete growth medium for 24 h (Relative Metabolic Activity). A second assessment of cytotoxicity was made by calculating the % attached cells from the live/dead cell counts and assigning a morphological grade according to the scale published in the ISO 10993-5 standard, where the 0–4 scale represents no, slight (not more than 20% round), mild (not more than 50% round), moderate (not more than 70% round) or severe cytotoxicity (nearly complete destruction of cell monolayers), respectively. To check that the cell cultures were free of mycoplasma contamination, the culture medium was saved and tested with positive and negative controls using the luminescent MycoAlert Plus mycoplasma detection kit (Lonza). All culture media samples tested negative for mycoplasmas. Statistical significance was determined using analysis of variance and Tukey–Kramer post-test.

### In vivo investigations

#### Biocompatibility testing in vivo

Miniaturized (1-cm diam.) smart dressing devices were tested in male SKH1 (hairless) mice using a splinted excisional wound healing model^50^. Mice were obtained at 8 weeks of age from Charles River Labs and were housed in groups of up to 5 per cage before surgery and singly after surgery. All animals had free access to standard chow and drinking water and were maintained on a 12-h light/dark cycle. Surgeries were performed when mice were 12–20 weeks of age (body weight 26–34 g) and followed a protocol that was approved by the Indiana University School of Medicine Animal Care and Use Committee.

All surgery, wound assessments and device perfusion was performed aseptically in an operating room of the animal care facility. Mice received pre-surgical analgesia by a subcutaneous injection of Buprenorphine SR LAB (1 mg/kg). Anesthesia was induced using 3% Isoflurane in 100% oxygen and maintained by 2% Isoflurane. Skin on the back of the mice was disinfected and rinsed three times using a 10% povidone-iodine solution and 70% ethanol, respectively. Two circular full-thickness wounds were created in the skin about the midline by punching through both sides of a fold of skin with a #8 biopsy punch. Two donut-shaped silicone splints (14 mm outer diameter, 10 mm inner diameter, 0.5-mm thick; Grace Bio-Labs) were affixed to the skin surrounding the wounds using tissue adhesive (Vetbond, 3 M) and 6 interrupted sutures (4-0 silk, Ethicon). The wound defects were filled with a collagen matrix (Dermal Regeneration Template, Integra Life Sciences) created by scraping the “dermal” collagen portion of the bilayer away from the silicone “epidermal” layer using a scalpel blade. For some mice, the collagen wound matrix was covered with a 1-cm diameter device containing Ru or KMnO_4_ ink or with a complete device that was perfused daily with H_2_O_2_ for up to 60 min. Perfused devices were left on the wounds overnight and then replaced for each subsequent H_2_O_2_ perfusion. Both wounds and devices were covered with a single piece of transparent dressing (Tegaderm, 3 M) and with an elastic wrap. Mice with wounds that were not oxygenated daily were brought to surgery twice per week to check for wound contamination and to photograph wounds for wound area calculations. Areas were calculated from digital photos (12 Megapixel) using ImageJ (NIH) and area values were calibrated using the area of the wound splint, which was considered to have a constant value. The wounds were biopsied following euthanasia on days 3, 7, or 14 post-surgery (*n* = 4 biopsies per treatment per time) and biopsies were fixed in 10% formalin prior to paraffin embedding, sectioning (20–25 μm) and staining with hematoxyin and eosin. Samples were then histologically assessed for the presence of neutrophils (acute inflammation) and macrophages (chronic inflammation) using a scoring system where a 0–4 scale represents none, low numbers, some, all over, or high numbers of cells.

#### Functionality testing in vivo

Wound oxygen measurements were performed in diabetic mice (BKS.Cg-*Dock7*^*m*^ +/+ *Lepr*^*db*^/J, stock #000642; Jackson Laboratories) using the smart dressing device, an optical sensor probe (FOSPOR, by Ocean Optics) and a phase fluorometer system (NeoFox, by Ocean Optics). The system was calibrated to 0% oxygen using a drop of sodium sulfite solution on a Ru(dpp)_3_Cl_2_ spot printed on parchment paper and to ambient oxygen (20.9%) using a device immediately prior to its placement onto the wound. The influx and efflux ports of the device were then connected to a syringe containing 3% H_2_O_2_ via a 3D printed connector and microtubing (Fig. 5a). The device was fit within the internal diameter of the wound splint and the probe was positioned perpendicular to and directly over the Ru(dpp)_3_Cl_2_ sensor and in gentle contact with the PDMS surface of the device. H_2_O_2_ was pumped through the device at a rate of 200 µL/h for up to 60 min and wound O_2_ measurements were recorded simultaneously using NeoFox Viewer (Ocean Optics).

## Supplementary information

Supplementary Information
