# Supplementary material for: Integrated sensing and delivery of oxygen for next-generation smart wound dressings
Source: Microsyst Nanoeng. 2020 May 18;6:46. doi: 10.1038/s41378-020-0141-7 (PMC8433317; doi:10.1038/s41378-020-0141-7)
Supplement: Supplementary file 1 — Supplementary Information [file 41378_2020_141_MOESM1_ESM.docx]

Integrated sensing and delivery of oxygen for next-generation smart wound dressings

M. Ochoa^ab^, R. Rahimi^ab^, J. Zhou^ab^, H. Jiang^ab^, C. K. Yoon^ab^, D. Maddipatla^c^, B. B. Narakathu^c^, V. Jain^bd^, M. Oscai^ab^, T. Morken^ab^, R. H. Oliveira^ab^, G. L. Campana^e^, M. A. Zieger^e^, R. Sood^e^, M. Z. Atashbar^c^, B. Ziaie^[[1]](#footnote-1)^*^abf^

**Supplementary Information**

**Table S1:** FUJIFILM Dimatix^™^ DMP 2831 Inkjet Printer Waveform Pattern Settings

| Waveform Pattern Settings | | | | | | | |
| --- | --- | --- | --- | --- | --- | --- | --- |
|  | | **Ru(dpp)_3_Cl_2_ dye Ink** | | | **KMnO_4_ Based Ink** | | |
|  | **Segment** | **Level (%)** | **Slew rate** | **Duration (µs)** | **Level (%)** | **Slew rate** | **Duration (µs)** |
| Jetting Waveform | 1 | 00 | 0.43 | 2.944 | 00 | 0.43 | 2.944 |
|  | 2 | 100 | 0.17 | 6.656 | 100 | 0.77 | 3.968 |
|  | 3 | 87 | 0.29 | 3.392 | 80 | 0.29 | 3.392 |
|  | 4 | 27 | 0.80 | 0.832 | 40 | 0.80 | 0.832 |
| Non-Jetting Waveform | 1 | 27 | 1.00 | 3.712 | 53 | 1.00 | 3.712 |
|  | 2 | 40 | 1.00 | 4.096 | 40 | 1.00 | 3.456 |
|  | 3 | 27 | 1.00 | 6.016 | 40 | 1.00 | 3.456 |


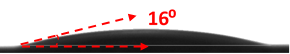

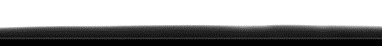


**Fig. S1 |** Contact angle of Ruthenium based ink, on Tyvek and parchment paper, measured using equilibrium contact angle method, showing relatively better wetting characteristics of parchment paper with a stable contact angle of ~35° for 60 seconds when compared to Tyvek paper with contact angle decreased from ~16° to <2° (complete spreading) in 5 seconds.

*
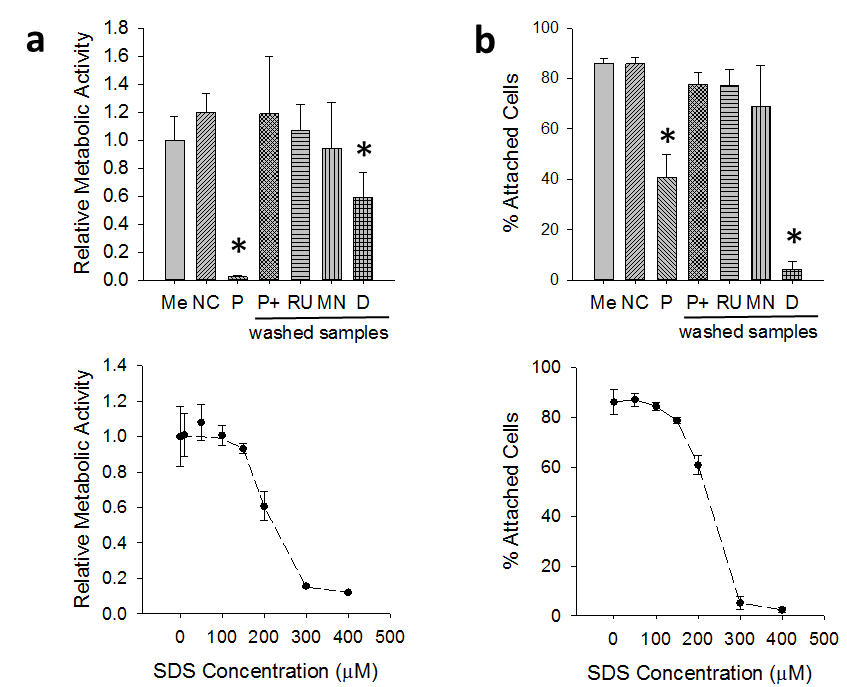
*

***
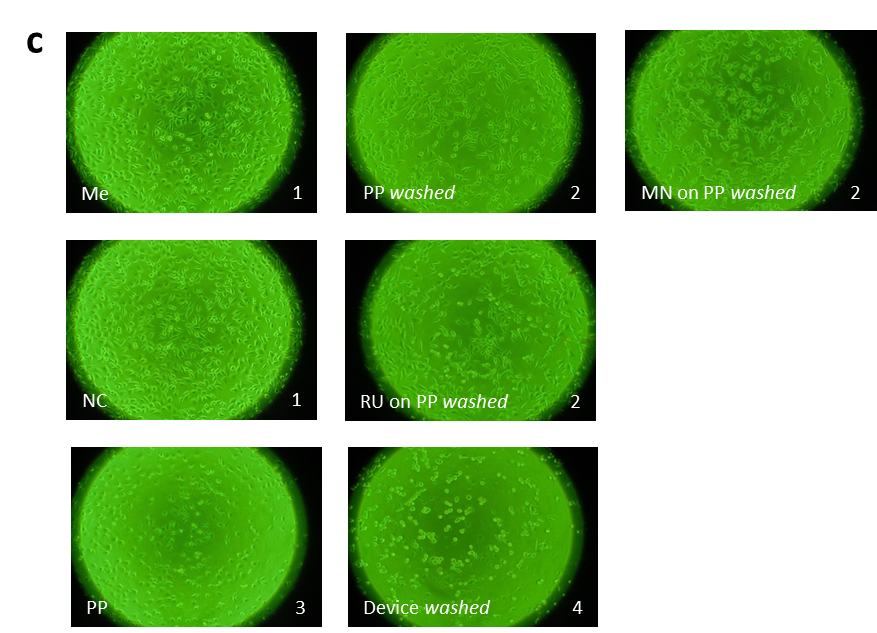
***

***
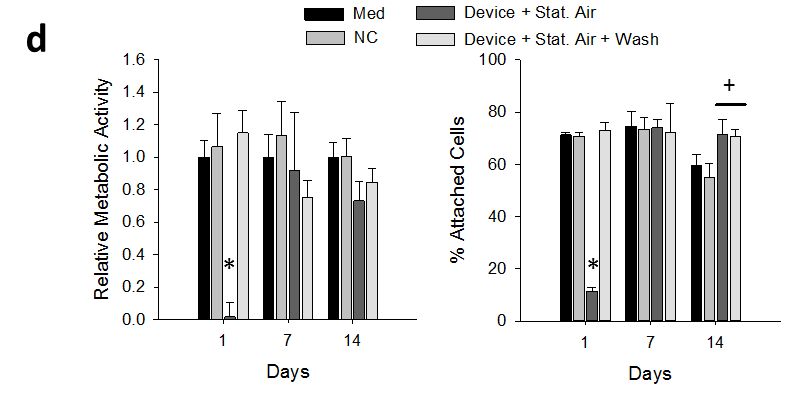
***

**e**

**Fig. S2 |** **Cytotoxicity of STERRAD® treated smart dressing devices and paper components.** (**a**) Relative Metabolic Activity measured by WST-1 assay (*above*). Values are means ± stdev of readings using extracts from four samples and *p<0.01 vs. all other treatments. *Below*, positive controls for cytotoxicity using SDS. (**b**) % Attached Cells (*above*) calculated from photomicrographs; values are means ± stdev of cell counts from six culture wells; *p<0.01 vs. all other treatments. *Below*, positive controls for cytotoxicity using SDS. (**c**) Photomicrographs of L-929 cells following 24 h treatment with extracts of devices, parchment paper or paper printed with Ru(dpp)_3_Cl_2_  or potassium permanganate ink; labelled with a morphological grade; *magnification* 33x. (**d**) Devices aerated for up to 14 days before extraction in culture medium and measurement of Relative Metabolic Activity (*left*) and % Attached Cells (*right*); values are means ± stdev from extracts of at least 2 devices; *p<0.01 vs. all other treatments and +p<0.01 vs. day 14 Med and day 14 NC. (**e**) Relative Metabolic Activity (*left*) and % Attached Cells (*right*) measured following treatment with extracts of devices perfused with H_2_O_2_ for 60 min, perfused with H_2_O_2_ followed by channel flushing with culture medium, or extracts of non-perfused devices. Me, cells treated with complete growth medium for 24 h; NC, extract made from low density polyethylene (negative cytotoxicity control); P, parchment paper; RU, parchment paper printed with Ru(dpp)_3_Cl_2_  ink; MN, parchment paper printed with potassium permanganate ink; D, complete 3-layer device; SDS, sodium dodecyl sulfate.

**Supplemental References**

1. Ochoa, M. Laser-processed parchment paper for fabrication of chronic wound dressings with selective oxygenation. (Purdue University, 2016).

1. * Corresponding authors: Professor Babak Ziaie (bziaie@purdue.edu) [↑](#footnote-ref-1)
